# Supplementary material for: Analysis of the Effects of the Vrn-1 and Ppd-1 Alleles on Adaptive and Agronomic Traits in Common Wheat (Triticum aestivum L.)
Source: Plants (Basel). 2024 May 23;13(11):1453. doi: 10.3390/plants13111453 (PMC11174496; doi:10.3390/plants13111453)
Supplement: Supplementary file 1 [file plants-13-01453-s001.zip › plants-2949670-supplementary.pdf]

Table S1. Molecular markers used in this study to specify alleles of *Vrn-1* and *Ppd-1* genes.

	Primers	Sequence (5' → 3')	Expected size, bp	Allele Variant	References
1	VRN1AF	GAAAGGAAAAATTCTGCTCG	965+876	Spring (*Vrn-A1a*)	[30]
	VRN1-INT1R	GCAGGAAATCGAAATCGAAG	714	Spring (*Vrn-A1b*)	
			734	Winter (*vrn-A1*)	
2	Intr1/C/F	GCACTCCTAACCCACTAACC	1068	Winter (*vrn-A1*)	[32]
	Intr1/AB/R	TCATCCATCATCAAGGCAAA			
3	Intr1/B/F	CAAGTGGAACGGTTAGGACA	709	Spring (*Vrn-B1*)	[32]
	Intr1/B/R3	CTCATGCCAAAAATTGAAGATGA			
4	Intr1/B/F	CAAGTGGAACGGTTAGGACA	1149	Winter (*vrn-B1*)	[32]
	Intr1/B/R4	CAAATGAAAAGGAATGAGAGCA			
5	Intr1/D/F	GTTGTCTGCCTCATCAAATCC	1671	Spring (*Vrn-D1*)	[32]
	Intr1/D/R3	GGTCACTGGTGGTCTGTGC			
6	Intr1/D/F	GTTGTCTGCCTCATCAAATCC	997	Winter (*vrn-D1*)	[32]
	Intr1/D/R4	AAATGAAAAGGAACGAGAGCG			
7	TaPpd-A1prodelF	CGTACTCCCTCCGTTTCTTT	338 (F/R2)	Insensitive (*Ppd-A1a*)	[50]
	TaPpd-A1prodelR3	AATTTACGGGGACCAAATACC	299 (F/R3)	Sensitive (*Ppd-A1b*)	
	TaPpd-A1prodelR2	GTTGGGGTCGTTTGGTGGTG			
8	TaPpd-B1proinF1	CAGCTCCTCCGTTTGCTTCC	620	Insensitive (*Ppd-B1a*)	[50]
	TaPpd-B1proinR1	CAGAGGAGTAGTCCGCGTGT	312	Sensitive (*Ppd-B1b*)	
9	Ppd-D1-F1	ACGCCTCCCACTACACTG	288 (F/R2)	Insensitive (*Ppd-D1a*)	[51]
	Ppd-D1-R1	GTTGGTTCAAACAGAGAGC	414 (F/R1)	Sensitive (*Ppd-D1b*)	
	Ppd-D1-R2	CACTGGTGGTAGCTGAGATT			
10	Ppd-B1_2ndcopy_F1	TAACTGCTCGTCACAAGTGC	425	Insensitive (*Ppd-B1c, CS*)	[51, 52]
	Ppd-B1_2ndcopy_R1	CCGGAACCTGAGGATCATC			
11	PpdB1_F25	AAAACATTATGCATATAGCTTGTGTC	994	Insensitive (*Ppd-B1c, CS*)	[52]
	PpdB1_R70	CAGACATGGACTCGGAACAC			
12	PpdB1_F3	CCAGGCGAGTGATTTACACA	223	Insensitive (*Ppd-B1a*)	[52]
	PpdB1_R36	GGGCACGTTAACACACCTTT			

Table S2. Pearson's correlations of the relevant principal components (PC) with determined quantitative and qualitative traits and correlation of these traits with each other.

	Vrn1	Vrn2	Vrn3	PPD-B-4	PPD-D	Awnless	Heading_Time_HT	Plant_Height_PH	Spike_Length_SL	Spikelets_Number_per_Spike_SNS	Tillering	Grains_Number_per_Spike_GNS	Grains_Weight_per_Spike_GWS	Fertility	PC1	PC2	PC3
Vrn1	1	-0,4	-0,62	-0,11	0,081	0,028	-0,8	-0,26	-0,28	-0,48	-0,21	-0,18	-0,2	0,029	-0,4	0,37	-0,58
Vrn2	-0,4	1	-0,47	-0,054	0,27	-0,18	0,46	-0,064	0,061	0,22	0,18	-0,0035	0,16	-0,076	0,089	-0,089	0,57
Vrn3	-0,62	-0,47	1	0,15	-0,31	0,13	0,38	0,31	0,22	0,28	0,049	0,18	0,05	0,037	0,31	-0,28	0,077
PPD-B-4	-0,11	-0,054	0,15	1	-0,43	0,15	-0,034	0,095	0,036	-0,02	0,041	-0,21	-0,07	-0,21	0,012	-0,37	-0,26
PPD-D	0,081	0,27	-0,31	-0,43	1	-0,86	-0,21	-0,65	-0,61	-0,3	-0,19	-0,16	-0,19	-0,02	-0,58	0,46	0,6
Awns	0,028	-0,18	0,13	0,15	-0,86	1	0,08	0,66	0,55	0,26	0,13	0,22	0,24	0,08	0,56	-0,31	-0,68
HT	-0,8	0,46	0,38	-0,034	-0,21	0,08	1	0,29	0,42	0,59	0,25	0,15	0,15	-0,09	0,47	-0,5	0,59
PH	-0,26	-0,064	0,31	0,095	-0,65	0,66	0,29	1	0,65	0,58	0,39	0,56	0,48	0,27	0,83	-0,14	-0,28
SL	-0,28	0,061	0,22	0,036	-0,61	0,55	0,42	0,65	1	0,72	0,48	0,54	0,6	0,23	0,88	-0,16	-0,05
SNS	-0,48	0,22	0,28	-0,02	-0,3	0,26	0,59	0,58	0,72	1	0,43	0,6	0,49	0,095	0,8	-0,2	0,35
Tillering	-0,21	0,18	0,049	0,041	-0,19	0,13	0,25	0,39	0,48	0,43	1	0,29	0,34	0,16	0,54	-0,05	0,22
GNS	-0,18	-0,0035	0,18	-0,21	-0,16	0,22	0,15	0,56	0,54	0,6	0,29	1	0,82	0,68	0,78	0,53	0,076
GWS	-0,2	0,16	0,05	-0,07	-0,19	0,24	0,15	0,48	0,6	0,49	0,34	0,82	1	0,57	0,75	0,47	0,066
Fertility	0,029	-0,076	0,037	-0,21	-0,02	0,08	-0,09	0,27	0,23	0,095	0,16	0,68	0,57	1	0,44	0,75	-0,072
PC1	-0,4	0,089	0,31	0,012	-0,58	0,56	0,47	0,83	0,88	0,8	0,54	0,78	0,75	0,44	1	-5,30E-16	7,80E-16
PC2	0,37	-0,089	-0,28	-0,37	0,46	-0,31	-0,5	-0,14	-0,16	-0,2	-0,05	0,53	0,47	0,75	-5,30E-16	1	7,30E-16
PC3	-0,58	0,57	0,077	-0,26	0,6	-0,68	0,59	-0,28	-0,05	0,35	0,22	0,076	0,066	-0,072	7,80E-16	7,30E-16	1

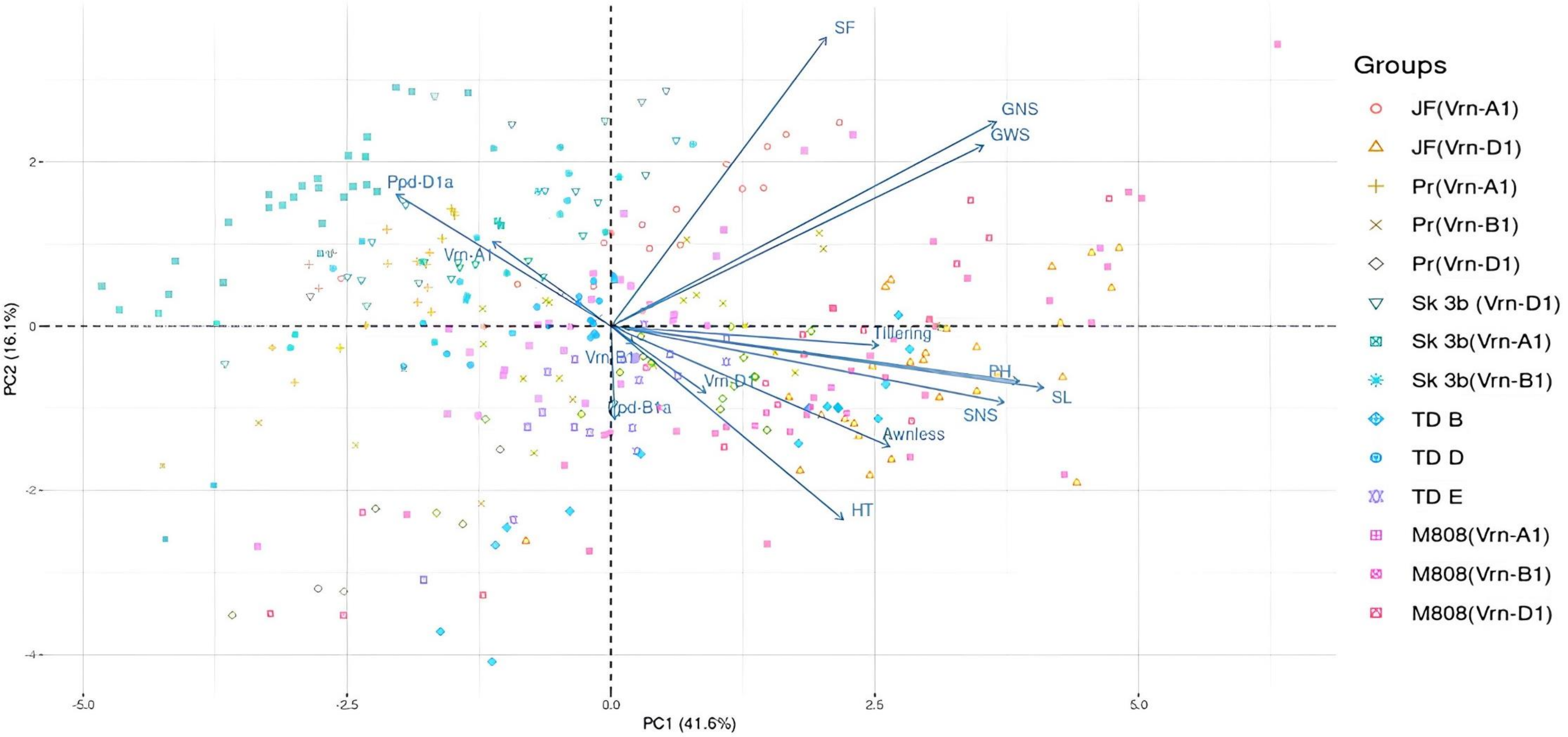

Figure S1. Bi-plots of the first two principal components PC1 vs. PC2 of wheat NILs based on distinct quantitative and qualitative traits (depicted as arrows). Percent of explained variance is depicted on the axes labels accordingly. Each dot represents an individual plant.

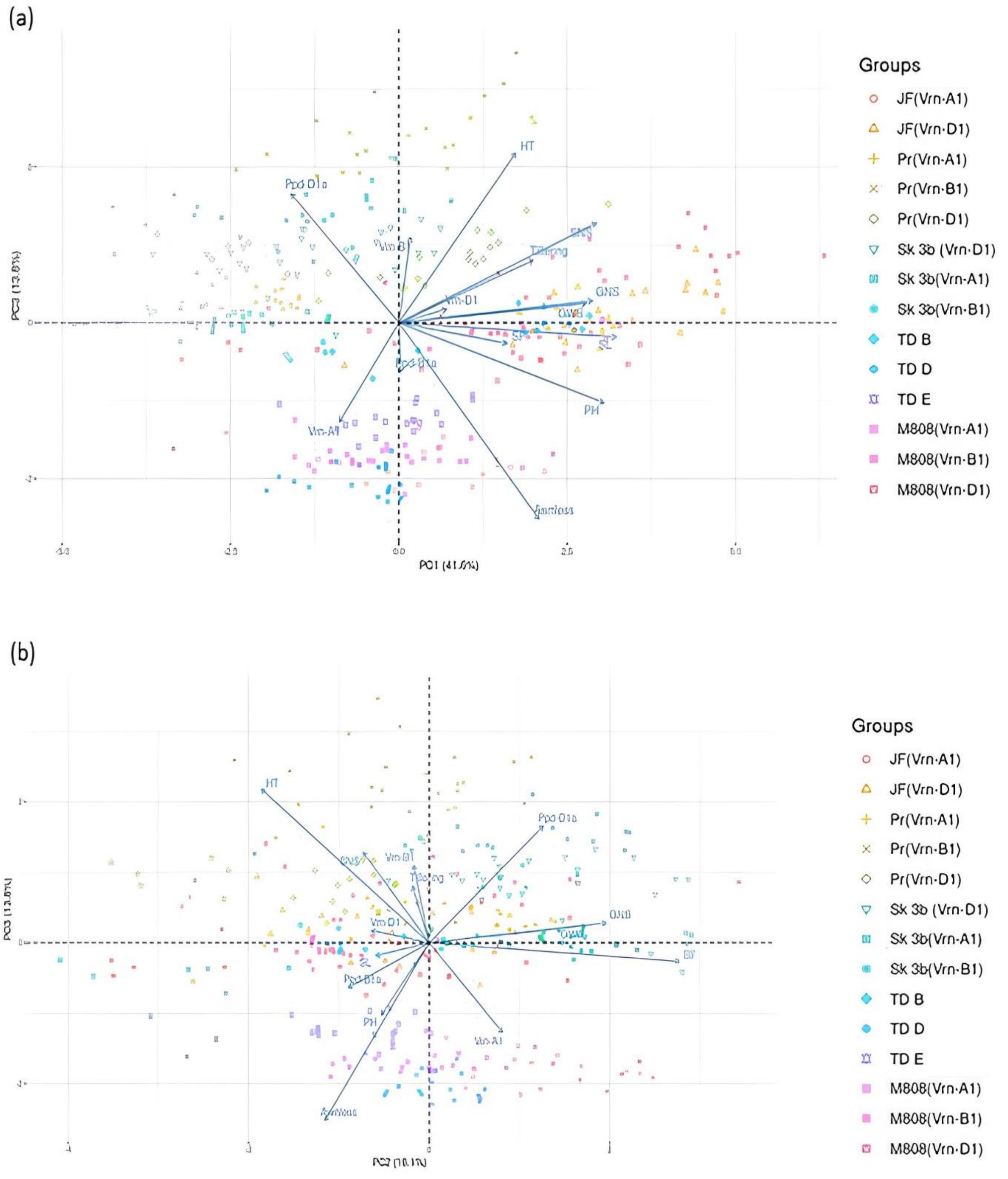

Figure S2. Bi-plots of the principal components, PC1 vs PC3 (a), PC2 vs PC3 (b) of wheat NILs based on distinct quantitative and qualitative traits (depicted as arrows). Percent of explained variance is depicted on the axes labels accordingly. Each dot represents an individual plant.
